# The Association between CAG Repeat Length and Age of Onset of Juvenile-Onset Huntington’s Disease

**DOI:** 10.3390/brainsci10090575

**Published:** 2020-08-20

**Authors:** Jordan L. Schultz, Amelia D. Moser, Peg C. Nopoulos

**Affiliations:** 1 Department of Psychiatry, Carver College of Medicine at the University of Iowa, Iowa City, IA 52242, USA; peggy-nopoulos@uiowa.edu; 2 Department of Neurology, Carver College of Medicine at the University of Iowa, Iowa City, IA 52242, USA; 3 Department of Psychology and Neuroscience, University of Colorado Boulder, Boulder, CO 80309, USA; amelia.moser@colorado.edu; 4 Department of Pediatrics, Carver College of Medicine at the University of Iowa, Iowa City, IA 52242, USA

**Keywords:** CAG, juvenile-onset Huntington’s disease, motor onset

## Abstract

There is a known negative association between cytosine–adenine–guanine (CAG) repeat length and the age of motor onset (AMO) in adult-onset Huntington’s Disease (AOHD). This relationship is less clear in patients with juvenile-onset Huntington’s disease (JOHD), however, given the rarity of this patient population. The aim of this study was to investigate this relationship amongst a relatively large group of patients with JOHD using data from the Kids-JOHD study. Additionally, we analyzed data from the Enroll-HD platform and the Predict-HD study to compare the relationship between CAG repeat length and AMO amongst patients with AOHD to that amongst patients with JOHD using linear regression models. In line with previous reports, the variance in AMO that was predicted by CAG repeat length was 59% (*p* < 0.0001) in the Predict-HD study and 57% from the Enroll-HD platform (*p* < 0.0001). However, CAG repeat length predicted 84% of the variance in AMO amongst participants from the Kids-JOHD study (*p* < 0.0001). These results indicate that there may be a stronger relationship between CAG repeat length and AMO in patients with JOHD as compared to patients with AOHD. These results provide additional information that may help to model disease progression of JOHD, which is beneficial for the planning and implementation of future clinical trials.

## 1. Introduction

Huntington’s disease (HD) is an autosomal dominant, neurodegenerative disorder that causes cognitive, behavioral, and motor symptoms [1]. An abnormal number of repeats (≥36) of cytosine–adenine–guanine (CAG) within the huntingtin gene causes the mutation that leads to HD. The negative relationship between the number of CAG repeats that a person has and the age of motor onset (AMO) has been well-established for patients with adult-onset HD (AOHD) [2,3,4,5,6]. However, given the rarity of juvenile-onset HD (JOHD), the relationship between CAG repeat length and AMO is less clear. In general, it is believed that the correlation between CAG repeat length and AMO increases in higher CAG repeat lengths [7,8,9], but these reports are limited by small sample sizes. A previous review paper utilized data from 15 patients with JOHD who had a CAG repeat length of >60 to demonstrate that the association between AMO and CAG seemed to be higher at CAG lengths of 60 to 80. However, at CAG repeat lengths above approximately 80, the association between AMO and CAG became much weaker [10]. In order to further investigate this phenomenon in a larger group, the same authors gathered patient data from seven separate case studies or case series of patients with JOHD and found 26 patients with a CAG repeat length of >80. In this cohort, CAG repeat length accounted for only 26% of the variance in AMO [10]. A recent systematic review was performed and gathered information on more than 200 patients with reported JOHD [11]. This cohort of patients included a wide range of CAG repeats from 39 to 265. The authors reported a Pearson’s correlation coefficient of −0.56 between CAG repeat length and the age of onset of clinical symptoms. In line with other studies, the strength of correlation seemed to weaken at longer CAG repeats [11]. Both of these large reviews are limited, however, by their reliance on a collection of data, rather than primary collection from a single cohort. Thus, the aim of the present study was to investigate the relationship between CAG repeat length and AMO amongst patients with JOHD, with the goal of providing additional clarity to this current gap in knowledge. We also leveraged data from the Enroll-HD platform and the Predict-HD study to compare the results of patients with JOHD to those of patients with AOHD.

## 2. Materials and Methods

### 2.1. Description of Data

**Figure 1:**
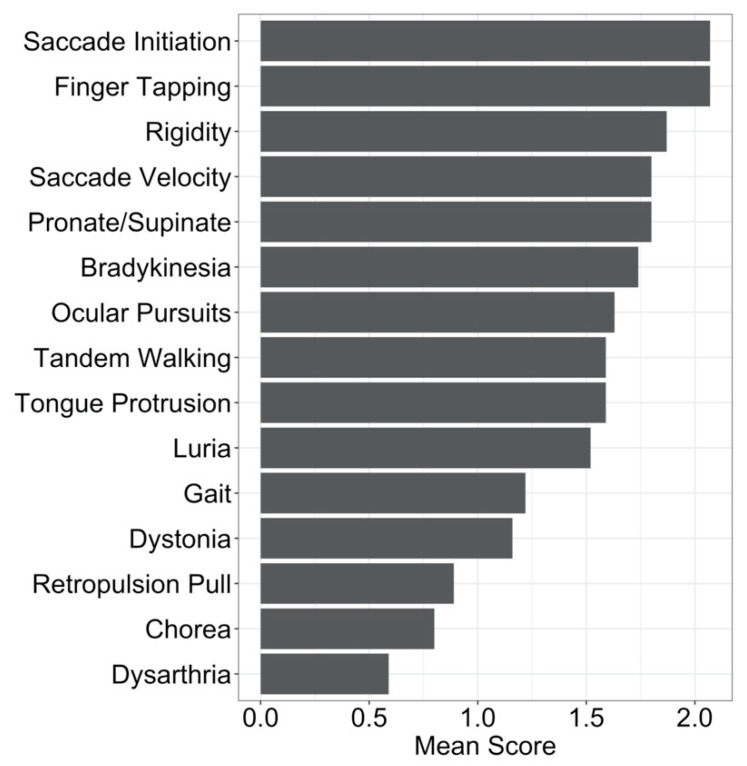
Mean scores on the various domains of the Unified Huntington’s Disease Rating Scale (UHDRS). Specifically, ocular pursuit score is the mean of horizontal and vertical pursuit scores; saccade initiation is the mean of the horizontal and vertical initiation scores; saccade velocity is the mean of the horizontal and vertical velocity scores; finger tapping is the mean of the right and left hand scores; pronate/supinate is the mean of the right and left hand scores; rigidity is the mean of the scores in the right and left arms; dystonia score is the mean scores from the trunk, right and left upper extremities, and right and left lower extremities; chorea score is the mean of maximal chorea scores from the face, buccal-oral-lingual scores, trunk, right and left upper extremities, and right and left lower extremities. All other scores include only one component and represent the mean of that domain.

We analyzed data from a large sample of participants enrolled in the Kids-JOHD studies (*N* = 27). These studies recruited participants from across the United States who were between the ages of 6 and 25 at the time of their first study visit and had a confirmed diagnosis of JOHD. To be enrolled, all participants were required to have already had molecular confirmation of HD and a clinical diagnosis of JOHD before traveling to the University of Iowa for assessment. To confirm that the participant was manifesting significant motor symptoms (meaning that they would meet criteria for a clinical diagnosis of JOHD), we utilized a combination of (1) detailed parental report of motor symptoms; (2) clinical evaluation by a pediatric neurologist at the University of Iowa; and (3) a Unified Huntington’s Disease Ratings Scale (UHDRS) Total Motor Score (TMS) [12] of at least 12 to consider a participant to be motor-manifest. Each subject was required to have all three to be considered motor-manifest. Although a TMS of 12 may be considered low, patients with JOHD often present with far greater hypokinetic symptoms than do those with AOHD, and the UHDRS may not be sufficiently sensitive to these as it was designed for AOHD. These criteria excluded 10 individuals enrolled in the Kids-JOHD study and represented subjects who were tested locally mainly based on family history or behavioral issues, rather than significant motor symptoms. Of note, the TMS derived from the UHDRS takes into account various motor symptoms beyond just chorea. At their baseline visit, the participants with JOHD demonstrated the highest scores in saccade initiation, finger tapping, and rigidity (Figure 1). Additionally, the summative chorea score was one of the areas where patients with JOHD had the lowest score. This is of particular importance to consider when confirming the diagnosis of JOHD, as symptoms other than chorea can often be present. Participants were also excluded if they had a history of brain surgery or significant head trauma. All participants and their guardians (if under 18) signed informed consent prior to enrolling in these studies, which were approved by the University of Iowa Institutional Review Board (IRB).

We also analyzed data from the Predict-HD study [13] and from the Enroll-HD platform [14]. Specifically, we included participants from both studies who received a motor diagnosis of HD during the study after the age of 21 and who had a CAG repeat length of less than 60. This was done to ensure that we were investigating only those participants with adult-onset HD (AOHD). Participant reporting of historical motor onset timing is included in the Enroll-HD platform, but this may be subject to significant recall bias. Participants must have had a motor exam conducted with a diagnostic confidence level (DCL) of less than four to be included in the analyses from Enroll-HD and Predict-HD. The age at which these participants had their first report of a diagnostic confidence level of four on the UHDRS [12] was considered the age of motor onset (AMO). There were 242 participants who received a motor diagnosis during the Predict-HD study and 782 participants in the Enroll-HD database.

### 2.2. Statistical Analysis

The primary analysis investigated the relationship between CAG repeat length and AMO amongst the JOHD participants using simple regression models. We used natural cubic splines to transform the independent variable (CAG repeat length) to investigate nonlinear relationships for the primary analysis. RStudio was used for all analyses, with a *p*-value of <0.05 considered statistically significant.

### 2.3. Institutional Ethical Approval

The University of Iowa institutional review board initially approved the study on 10/13/2011 (IRB # 201109879). For participants younger than 18 years, parents or guardians provided written consent and children provided written assent. For participants who were 18 years or older, participants provided written consent.

## 3. Results

**Figure 2:**
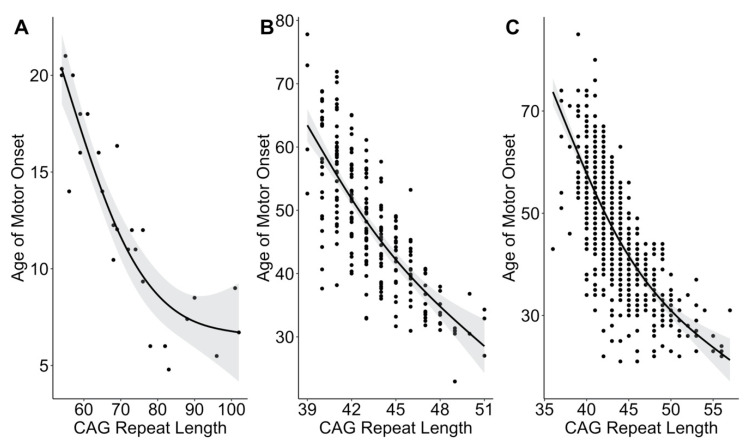
Cytosine–adenine–guanine (CAG) repeat length significantly predicts the age of motor onset in (**A**) patients with juvenile-onset Huntington’s disease (JOHD) from the Kids-JOHD study, (**B**) patients with adult-onset Huntington’s Disease (AOHD) from the Predict-HD study, and (**C**) patients with AOHD from the Enroll-HD study. Black lines show the predicted regression lines, and the gray ribbons display the 95% confidence intervals.

The participants from the Kids-JOHD study had a significant nonlinear relationship between AMO and CAG repeat length (*R^2^* = 0.84, *p* = 2.63 × 10^−10^) (Figure 2A). As has been reported previously, we also observed significant nonlinear relationships between AMO and CAG repeat length using data from participants with AOHD from the Predict-HD and Enroll-HD studies. From Predict-HD, the *R^2^* value was 0.59 (*p* = 2.2 × 10^−16^) (Figure 2B). The results from participants from Enroll-HD closely matched those observed in Predict-HD. Specifically, the R^2^ value was 0.57 (*p* = 2.2 × 10^−16^) (Figure 2C).

## 4. Discussion

In the present analysis, we demonstrated that the CAG repeat length accounted for over 80% of the variance in AMO amongst patients with JOHD. This was substantially higher than in either of the AOHD analyses conducted, which showed CAG repeat length accounting for 59% of the variance (Predict-HD sample) and 57% of the variance (Enroll-HD). These results support previous reports demonstrating potentially increased predictive power of higher CAG repeat lengths [7,8,9]. Of note, previous reports have demonstrated that the predictive power of CAG repeat length on AMO seems to decrease at the highest CAG repeats of approximately 80 or above [10,11]. Our current cohort only included seven participants with a CAG repeat length above 80. Therefore, we did not have sufficient data to formally analyze whether or not the relationship between CAG and AMO weakens at higher CAG repeat lengths. However, informally, our results seem to confirm these previous reports. In Figure 2A, it seems as though there is a strong, linear relationship between CAG repeat length and AMO in those participants with a CAG repeat length of <80. There seems to be a bend in the regression line at approximately a CAG repeat length of 80, where the line begins to flatten out. This same shape was seen in previous reports with larger numbers of patients [10,11]. This may be due to a floor effect in the ability of the CAG repeat length to predict AMO. Specifically, it may be possible that neurodegenerative changes occur over the course of 3–5 years. Therefore, the earliest possible AMO may be approximately five years old, regardless of CAG repeat length. This is only a hypothesis, though. Another possible explanation for the weakened relationship between CAG and AMO at CAG repeats above 80 may be related to the role that the huntingtin protein plays in neurodevelopment [15,16]. Neurodevelopmental changes have been reported to be more prominent at higher CAG repeats in patients with AOHD [15]. At higher CAG repeats, it is possible that the neurodevelopmental aberrations play a major role in the AMO in addition to neurodegeneration. This likely leads to significant difficulty in determining when their actual AMO is, likely resulting in a significant amount of heterogeneity in age of diagnosis.

One possible explanation for why higher CAG repeat lengths (>60) may explain more of the variance in AMO is that longer CAG repeat lengths may play a greater role in the development of pathologic changes that impact the onset of disease. Another consideration is that it is known that environmental exposures may modify disease onset in patients with AOHD [17,18,19]. Patients with JOHD may not have the same opportunity to be exposed to particular environmental factors. Therefore, their AMO is more closely linked to CAG repeat length alone and not the additional impact of environmental factors. Genetic modifiers of disease onset have also been identified in patients with AOHD [20,21]. These studies rely on large numbers of patients to identify genetic modifiers that may impact disease onset in AOHD. It cannot be ruled out that specific genetic modifiers exist that could impact the AMO in JOHD that have not been identified, given the rarity of this patient population.

One of the largest previous reports of the relationship between CAG repeat length and AMO amongst patients with JOHD utilized data from the Italian Huntington’s Disease Databank, which retrospectively collected data from patients at two separate institutions and only includes 15 patients with a CAG repeat length of >60 [10,22]. This same review gathered data from seven separate case reports and case series and identified 26 patients with more than 80 CAG repeats [10]. Given the means by which these data were collected, the conclusions drawn in the resulting review may be seen as preliminary, as they are not the result of primary data collection. However, using the present large dataset of patients with JOHD, we are now able to confirm these previous findings showing that the strength of correlation between CAG and AMO is greater in JOHD, predicting about 84% of the variance in AMO [10].

There are important limitations to this study. First, despite being one of the largest studies of JOHD in the world, the number of patients is still relatively small. Second, we did not implement natural logarithmic transformations of our data, which has been recommended in previous studies investigating CAG and AMO [23]. We opted to not use natural logarithmic transformations of the data because doing so appeared to lead to disproportionate variance across the groups, which would lead to heteroscedasticity in the data. Lastly, as mentioned previously, the diagnosis of motor symptoms in children with the longest CAG repeats can be quite difficult and subject to bias and variability.

## 5. Conclusions

This study has provided further confirmation that CAG repeat length significantly predicts AMO in patients with JOHD. In fact, more than 80% of the variance of AMO was explained by CAG repeat length. This provides additional information that allows for more accurate modeling of JOHD, which is critical for future clinical trials.
